# Coccoid *Helicobacter pylori* in patients with obesity: an immunohistochemical study

**DOI:** 10.1007/s00428-025-04042-4

**Published:** 2025-02-01

**Authors:** Chen Mayer, Daniel Hillel, Iris Barshack, Michael Schvimer

**Affiliations:** 1 Pathology Institute, Sheba Medical Center, Tel Hashomer, Israel; 2 Faculty of Medicine, Tel-Aviv University, Tel Aviv, Israel

**Keywords:** *Helicobacter pylori*, Obesity, Coccoid form, Antibiotic eradication

## Abstract

*Helicobacter pylori* (HP) is a Gram-negative bacterium that infects approximately fifty percent (50%) of individuals worldwide. The coccoid form of HP, a dormant state with altered morphology, has been associated with persistent infections and antibiotic resistance. This study aimed to investigate the prevalence of the coccoid form of HP in patients living with obesity. Sleeve gastrectomy specimens from obese patients and gastric biopsies from non-obese individuals were analyzed. Immunohistochemistry (IHC) staining and histopathological examination were performed to identify and quantify the coccoid forms of HP. Statistical analysis was conducted to compare the results between the two groups. The study included 53 obese patients and 62 non-obese individuals. The percentage of coccoid forms of HP was significantly higher in obese patients compared to non-obese individuals (median 50% vs. 10%, *p* < 0.001). Type of gastritis was also significantly different between the groups. Obese patients exhibited a higher prevalence of the coccoid form of HP in their gastric mucosa. This finding suggests that the gastric microenvironment in obesity may favor the formation of the coccoid form, potentially impacting the colonization and pathogenicity of HP. The higher prevalence of the coccoid form in obese patients has important clinical implications, as it is more resistant to antibiotics and difficult to eradicate. Alternative treatment strategies may be necessary to effectively manage HP infections in this population. Furthermore, the presence of the coccoid form may increase the risk of HP-associated diseases in obese individuals. Further research is needed to elucidate the underlying mechanisms and explore novel treatment approaches for HP infection in the context of obesity.

## Introduction

*Helicobacter pylori* (HP) is a Gram-negative bacterium that infects approximately 50% of individuals worldwide, with even higher rates of infection in developing countries [1]. Since first culturing *Helicobacter pylori* decades ago, the rate of HP acquisition in industrialized countries has decreased substantially. Although linked to various diseases, these occur only in a small percentage of infected individuals. It has also been suggested that in some hosts, HP may even play a beneficial role in human health [2].

Most *Helicobacter* infections are known to be acquired during childhood and are significantly influenced by geographical context and living conditions [3]. In developed countries, infection rates among children are between 1 and 12%, reaching only 30–50% in adults [1]. The continuous increase with age is mostly related to the cohort effect, reflecting delayed exposure to the bacteria. The main routes of HP transmission are person-to-person by oral-oral or fecal–oral routes, making hygiene and sanitation important risk factors for infection, in addition to socioeconomic status.

*Helicobacter pylori* is associated with several gastrointestinal diseases, including chronic gastritis, peptic ulcers, gastric carcinoma, mucosa-associated lymphoid tissue (MALT) lymphoma, and other cancers [4]. However, in most human hosts, it does not induce evident negative effects. The pathogen causes persistent infection and deregulation of host functions, including the immune reaction. In the stomach, it uses its motility, excreted chemoreceptors, and ability to adhere to outer membrane proteins to reach the protective mucus layer at the surface of the gastric mucosa [5].

Like other microorganisms, HP is also able to modify its morphology in order to survive adverse environmental conditions such as exposure to antibiotics, changes in temperature, pH, or oxygen tension [6]. Under these conditions, the pathogen can alter its state into an inactive “coccoid” form, a “viable but non culturable” (VBNC) state. In this dormant state, the bacteria lose their classical spiral shape, shown to be crucial for gastric colonization and mucus penetration. Despite its hibernating status, the bacteria maintain low urease activity and expression of virulent urease encoding genes, detectable by polymerase chain reaction (PCR) [7]. In fact, this coccoid form continues to express all major virulent genes—ureA, ureB, hpaA, vacA, cagA, cagE, and BabA [8]. This coccoid form can persist for over a year, and when environmental conditions become favorable, the bacteria can re-acquire their spiral shape along with its virulence and culturability.

**Fig. 1:**
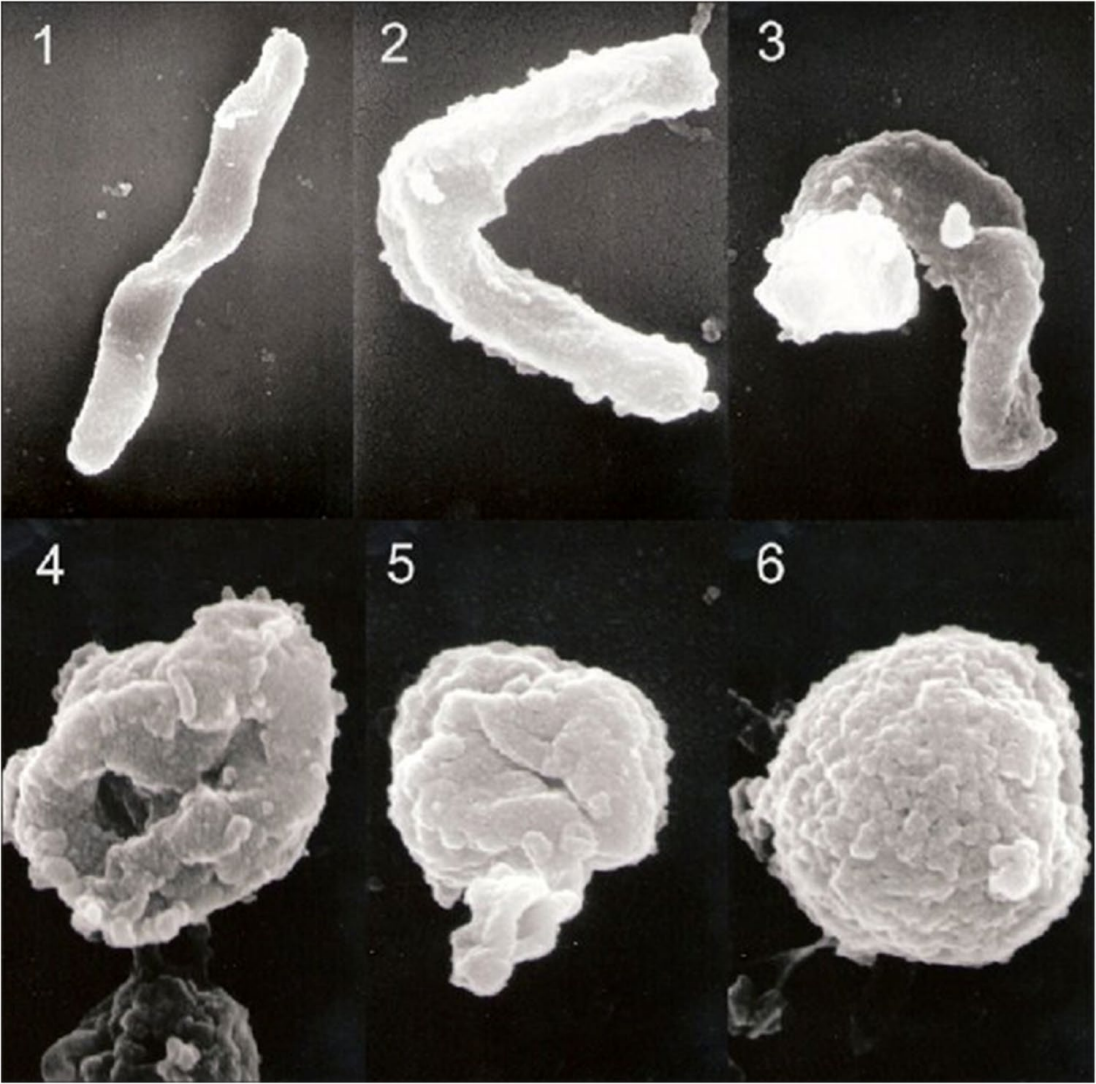
Conversion from the bacillary form of *H. pylori* to the coccoid form [10]

Electron microscopy preformed during the conversion stage from bacillary spiral shape to the coccoid form reveals the intermediate shapes of the bacterium in this process [Fig. 1]. Initially, the protoplasmic matrix shrinks and the periplasm increases on the side opposite the flagella basal complex. These changes lead to stretching of the cell and accumulation of matrix at the cell periphery, resulting in a U/C-shaped cell [9]. The change into coccoid form produces two sub-types: subtype A with irregular sides and a rugged surface, termed “the dead cell,” and subtype B with a smooth surface, termed “the living cell.”

In the human gastric mucosa, HP can turn coccoid with exposure to anti-secretory or anti-bacterial drugs. This change can also occur due to the accumulation of toxic metabolic products, such as reactive oxygen species or the presence of specific pyrimidine nucleotides. It has also been reported that the percentage of coccoid HP is higher in the duodenum than in the stomach [10], perhaps due to the unfavorable conditions. The coccoid transformation process may take only a few hours after adhesion to the surface of gastric epithelium. [11]

As stated above, the coccoid form of HP is non-culturable and difficult to detect with conventional methods. Electron microscopy may directly visualize the coccoid forms, as demonstrated by Willén et al. [12]. Fluorescent in situ hybridization is a relatively sensitive tool and can be used for detection in water samples [13]. Overall, molecular-based techniques are still considered the gold standard for detection. PCR and real-time PCR are the most widely used, as they can detect the organism even in low numbers.

Immunohistochemistry (IHC) is also used for detection of HP in clinical settings, while some pathologists still rely on H&E-stained sections as sufficient for the diagnosis of *Helicobacter* infection. Other than routine H&E-stained slides, Giemsa and silver staining are also helpful in identifying HP bacteria on histologic sections. IHC is a reliable technique to identify Helicobacter and differentiate coccoid HP from similar morphologic entities, such as different bacterial cocci, fungal spores and rare pathogens such as cryptosporidia [14].

In this study, we show the increased prevalence of the coccoid form of *Helicobacter pylori* in the stomachs of patients suffering from obesity. To do so, we used H&E staining together with bacterium-specific immunohistochemical staining to describe and quantify the percentage of these coccoid forms in sleeve gastrectomy specimens.

## Methods

### Sample collection

All sleeve gastrectomy cases performed at the Sheba Medical Center between 2020 and 2022 were retrieved from the pathology archives, along with BMI values and demographic information from medical files. In Israel, bariatric surgery is only approved for patients with a BMI > 40 or patients with a BMI > 35 with obesity-related illnesses such as hypertension and diabetes. Before such surgeries, a single dose of antibiotic prophylaxis is administered within 60 min prior to the procedure, which is not expected to significantly alter the gastric Helicobacter state.

Selecting the appropriate patient group for comparison posed a challenge, as it was difficult to procure a group of healthy patients with low BMI who had undergone stomach biopsy or resection. Given the tissues available to us, three options were considered: First, unselected gastric biopsies with no pathologic changes. Due to the very low prevalence of HP in normal gastric biopsies, the number of cases required to obtain informative data was prohibitive. Second, partial gastrectomies from Whipple procedures, performed due to malignancy. However, the major illness and oncologic treatments received would pose a significant confounder that could not be dismissed.

Finally, endoscopic biopsies signed out as *Helicobacter pylori* positive. This group was ultimately selected. Patients over the age of 60 and those with gastric malignancy or atrophic gastritis were excluded. These specimens were also from 2020 to 2022 and retrieved from the archives of the Pathology Institute of Sheba Medical Center. Initial tests for the diagnosis of HP were at the discretion of the pathologist signing out the case.

Cases were reviewed in a blinded manner by a single oriented pathologist using both physical and digital slides. Images were scanned at 40 × magnification using the Philips IntelliSite Ultra-fast scanner (Philips Digital Pathology Solutions, Best, Netherlands), capable of digital magnification up to 100x.

Cases were reviewed for type of infection, type of gastric mucosa, findings of chronic or active inflammation, and the estimated percentage of the coccoid form of HP on H&E and IHC-stained slides.

### Fixation and staining

Tissues were processed according to standard pathological procedures. Tissues were fixed in buffered formalin, embedded in paraffin, sectioned at 3.5 microns and routinely stained with H&E. For immunohistochemical studies, 4-μm-thick sections were prepared from FFPE blocks and stained using “215A-76 Cell Marquee Helicobacter Pylori” antibody.

### Statistical analysis

Age and coccoid percentage distributions were evaluated using a histogram. Since both variables were not normally distributed, they were described using the median and interquartile range (IQR). The chi-square test and Fisher’s exact test were used to compare categorical variables between the groups. The Mann–Whitney *U* test was used to compare continuous variables between the groups. Spearman's rank correlation coefficient was used to investigate the correlation between age and coccoid percentage. All statistical tests were two-sided. A *p*-value < 0.05 was considered statistically significant.

IBM SPSS 26 was used for all statistical analysis (IBM Corp. Released 2019. IBM SPSS Statistics for Windows, Version 26.0. Armonk, NY: IBM Corp).

### Ethics approval

The study was approved by the Ethics Committee, IRB 9430–22-SMC, in accordance with the Declaration of Helsinki. IRB 9430–22-SMC waived the requirement for informed consent.

## Results

We initially extracted 91 cases from patients who underwent a sleeve gastrectomy procedure. After excluding cases that showed no inflammation and no presence of *H. pylori* on immunohistochemistry, we were left with 53 cases in the obese group.

In the control group, we started with 77 cases initially signed out as HP positive. Of these, 15 cases were negative on immunohistochemistry for HP that was subsequently performed for the purpose of this study. This left us with 62 cases in the non-obese group.

In total, 115 individuals were included in the study, with approximately half (46.1%) being individuals with severe obesity (BMI > 35) and the other half not suffering from obesity. The median age was 38.4 years (IQR 21.9–49.2). Patient characteristics are summarized in Table 1. The type of gastritis was significantly different between the groups, with 74.2% of the non-obese group showing chronic active inflammation, while all obese patients exhibited chronic inactive inflammation (*p* < 0.001). There was no significant difference between groups in type of inflammation (*p* = 0.155) or age (*p* = 0.462).

**Table 1:** Patient characteristics summary

Variable	Non-obese *n* = 62	Obese *n* = 53	All *n* = 115	*p*-value
Age (IQR)	38.12 (28.7)	36.53 (27.04)	37.38 (27.29)	0.462
Gastritis				< 0.001
None	0 (0.0%)	1 (1.9%)	1 (0.9%)	
Chronic	16 (25.8%)	52 (98.1%)	68 (59.1%)	
Chronic active	46 (74.2%)	0 (0.0%)	46 (40.0%)	
Mucosa type				< 0.001
Oxyntic	19 (30.6%)	53 (100.0%)	72 (62.6%)	
Antral	28 (45.2%)	0 (0.0%)	28 (24.3%)	
Both	15 (24.2%)	0 (0.0%)	15 (13.0%)	
Inflammation				0.155
None	0 (0.0%)	1 (1.9%)	1 (0.9%)	
Superficial	44 (71.0%)	43 (81.1%)	87 (75.7%)	
Superficial and deep	18 (29.0%)	9 (17.0%)	27 (23.5%)	
Coccoid percentage (IQR)	8.7% (10)	55.28% (65)	30.17% (50)	< 0.001

**Fig. 2:**
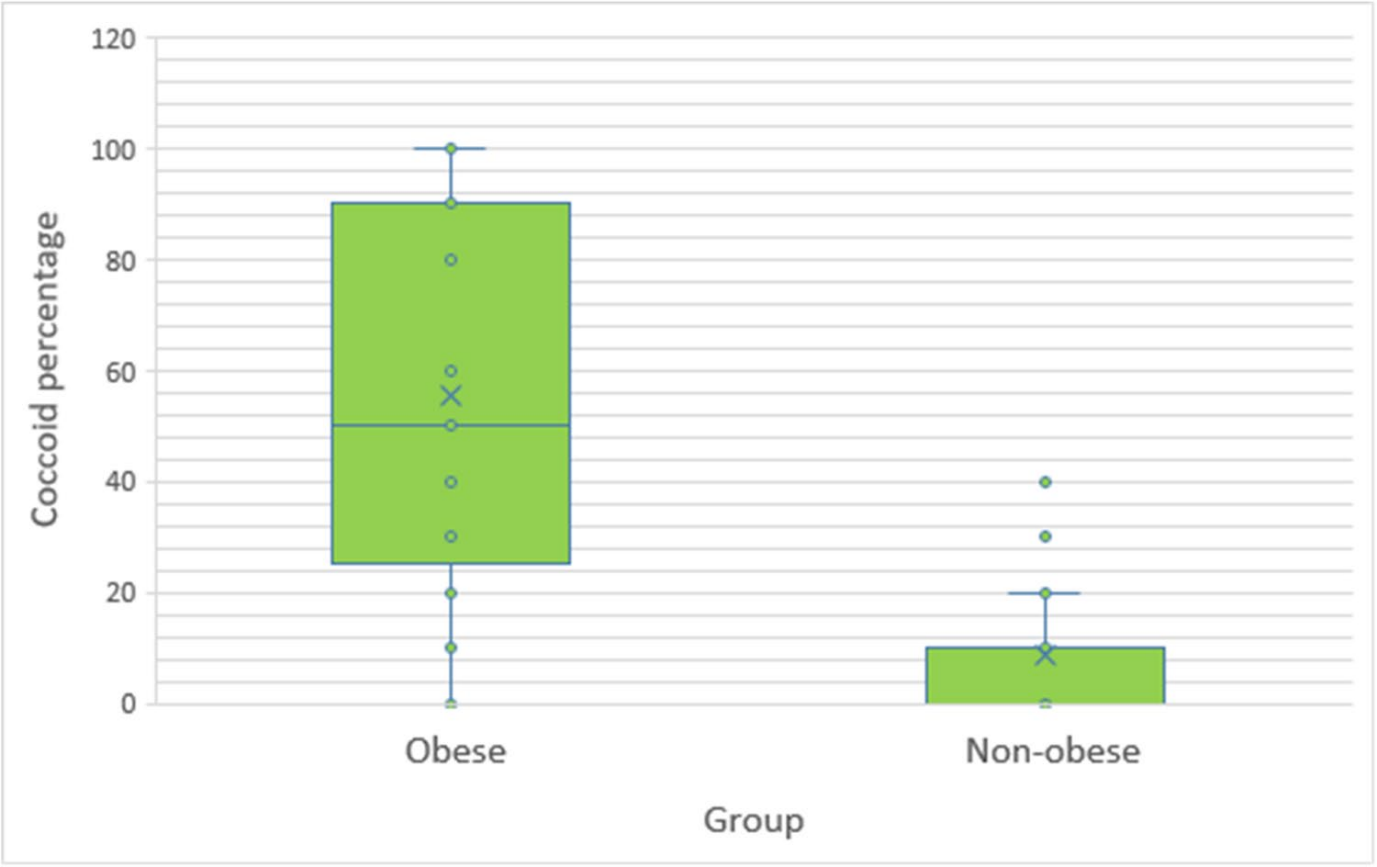
Box and whiskers plot demonstrating the distribution of the coccoid percentage in both groups

The coccoid percentage was significantly higher in people living with obesity (median 50, IQR 25–90) compared to the non-obese group (median 10, IQR 0–10) (*p* < 0.001). [See Table 2 and Fig. 2].

**Table 2:** Coccoid percentage

Number of patients		Coccoid percentage
Number of patients	Number of patients	
57	13	0–25
6	19	26–50
0	1	51–75
0	20	76–100

There was no significant association between age and coccoid percentage (*r* = − 0.91, *p* = 0.334). Correlation between age and coccoid percentage was also evaluated separately in each group, with no significant correlation found in either group. Additionally, there was no significant association between coccoid percentage and type of inflammation (*p* = 0.189).

## Discussion

The presence of the coccoid form of *Helicobacter pylori* has been implicated in persistent infections and antibiotic resistance. Our study has demonstrated that obese patients exhibit a higher percentage of the coccoid form of *H. pylori*, which may contribute to the difficulty in treating *H. pylori* infections in this population.

The gastric microenvironment in obese individuals may favor the formation of the coccoid form, although the exact mechanism underlying this observation is not yet clear. This change in the gastric microenvironment may affect the colonization and pathogenicity of *H. pylori*.

The higher prevalence of the coccoid form of *H. pylori* in obese patients has important clinical implications. The coccoid form of HP has been shown to be more resistant to antibiotics and more difficult to eradicate than the helical form. Therefore, alternative treatment strategies may be required to effectively treat HP infection in patients living with obesity. The use of novel antibiotics, combination therapy and probiotics may be beneficial in the treatment of *H. pylori* infection in this population. Weight management may also have a beneficial effect in the treatment and prevention of HP-associated diseases in overweight individuals.

Another important implication of our study is the potential impact of the coccoid form of HP on the overall health of obese individuals. Chronic HP infection has been associated with various health conditions, including gastric cancer, peptic ulcer disease and gastric lymphoma. The higher prevalence of the coccoid form of HP in these patients may increase their risk of developing these conditions, with obesity alone already related to many of these diseases.

A limitation of our study is the selection of the control group. As detailed, we were hindered by the availability of tissues for assessment, and therefore could not compare the sleeve gastrectomy specimens to a truly parallel group of patients. In the selection process, our main goal was to determine the percentage of coccoid forms within the *Helicobacter* groups, rather than the overall prevalence of *Helicobacter* in the tissue. Despite this limitation, we believe that the significantly distinct differences between the groups further prove our hypothesis, as we expected a higher percentage of the coccoid forms in the heterogenic non-obese group we selected, without obtaining information regarding previous treatments and associated symptoms.

There is also a difference in mucosal type among the two groups. While the obese patient group consists entirely of oxyntic mucosa biopsies due to the nature of the surgical procedure, the non-obese group consists of a mixed group of oxyntic and antral gastric mucosa. This difference further emphasizes our results, as *H. pylori* is known to be found in higher amounts in antral mucosa. In the non-obese group, most cases with over 10% of the coccoid form were found within antral mucosa (10 out of 14 cases).

The gastritis in obese patients undergoing bariatric surgery is typically a mild, chronic inactive gastritis involving the superficial mucosa, predominantly lymphoplasmacytic, lacking reactive lymphoid aggregates and dominated by the coccoid form of HP. This contrasts with the gastritis seen in the general population with HP infection, which is more often chronic active, lymphoplasmacytic, with reactive lymphoid aggregates, and may infrequently harbor the coccoid form of HP in significant numbers.

During the examination of the slides, we noted that using digital pathology to identify and categorize HP is nearly impossible with a 2-dimensional scanner due to the size and spatial complexity of the organism. Even with immunohistochemical scanned slides, it was not possible to differentiate the various forms of *H. pylori*. After several attempts at scanning the slides by different methods, we ultimately examined all cases by light microscopy.

In clinical practice, the coccoid form of HP may be underdiagnosed due to the lack of familiarity of many pathologists with this form of HP infection, along with the inherent difficulty of identifying coccoid bacteria present in small numbers within biopsies showing relatively mild gastritis. Guidelines for the routine use of IHC staining for HP support this notion [15].

At our facility, it is not customary to perform upfront immunohistochemistry for *H. pylori* on all cases, as is the protocol in some institutes around the world. It is not surprising, therefore, that we found false positive cases within our archives when IHC was performed for the purpose of this study. While some guidelines refrain from recommending upfront IHC, following our results, we have become more lenient in ordering immunohistochemical stains to distinguish these bacteria and plan to revisit this subject in the future.

## Conclusions

Our study highlights the need for personalized medicine in the treatment of HP infection. The higher prevalence of the coccoid form of HP in obese individuals suggests that conventional antibiotic regimens may not be effective in this population. Therefore, personalized treatment strategies, such as the use of tailored antibiotic regimens, combination therapy, and probiotics, may be necessary to effectively treat this infection.

## Data Availability

The data that support the findings of this study are available from the corresponding author (CM), upon reasonable request.
